# ADAM: Analysis of Discrete Models of Biological Systems Using Computer Algebra

**DOI:** 10.1186/1471-2105-12-295

**Published:** 2011-07-20

**Authors:** Franziska Hinkelmann, Madison Brandon, Bonny Guang, Rustin McNeill, Grigoriy Blekherman, Alan Veliz-Cuba, Reinhard Laubenbacher

**Affiliations:** 1 Virginia Bioinformatics Institute, Virginia Tech, Washington Street, MC 0477, Blacksburg, VA 24061, USA; 2 Department of Mathematics, Virginia Tech, 460 McBryde, Blacksburg, VA 24061-0123, USA; 3 Department of Mathematics, The University of Tennessee, 227 Ayres Hall, 1403 Circle Drive, Knoxville, TN 37996-1320, USA; 4 Department of Mathematics, Harvey Mudd College, 301 Platt Boulevard, Claremont, CA 91711-5901, USA; 5 Department of Mathematics and Statistics, University of North Carolina - Greensboro, 116 Petty Building, Greensboro, NC 27402-6170, USA

## Abstract

**Background:**

Many biological systems are modeled qualitatively with discrete models, such as probabilistic Boolean networks, logical models, Petri nets, and agent-based models, to gain a better understanding of them. The computational complexity to analyze the complete dynamics of these models grows exponentially in the number of variables, which impedes working with complex models. There exist software tools to analyze discrete models, but they either lack the algorithmic functionality to analyze complex models deterministically or they are inaccessible to many users as they require understanding the underlying algorithm and implementation, do not have a graphical user interface, or are hard to install. Efficient analysis methods that are accessible to modelers and easy to use are needed.

**Results:**

We propose a method for efficiently identifying attractors and introduce the web-based tool Analysis of Dynamic Algebraic Models (*ADAM*), which provides this and other analysis methods for discrete models. *ADAM *converts several discrete model types automatically into polynomial dynamical systems and analyzes their dynamics using tools from computer algebra. Specifically, we propose a method to identify attractors of a discrete model that is equivalent to solving a system of polynomial equations, a long-studied problem in computer algebra. Based on extensive experimentation with both discrete models arising in systems biology and randomly generated networks, we found that the algebraic algorithms presented in this manuscript are fast for systems with the structure maintained by most biological systems, namely sparseness and robustness. For a large set of published complex discrete models, *ADAM *identified the attractors in less than one second.

**Conclusions:**

Discrete modeling techniques are a useful tool for analyzing complex biological systems and there is a need in the biological community for accessible efficient analysis tools. *ADAM *provides analysis methods based on mathematical algorithms as a web-based tool for several different input formats, and it makes analysis of complex models accessible to a larger community, as it is platform independent as a web-service and does not require understanding of the underlying mathematics.

## Background

Mathematical modeling is a crucial tool in understanding the dynamic behavior of complex biological systems. In addition to the popular ordinary differential equations (ODE) models, discrete models are now increasingly used for this purpose [1-3]. Model types include (probabilistic) Boolean networks, logical networks, Petri nets, cellular automata, and agent-based (individual-based) models, to name the most commonly found ones [4-9]. While discrete models tend to be more intuitive than those based on differential equations, they do not have the broad range of mathematical analysis tools available that have been developed for ODE models. For small models, exhaustive enumeration of all possible state transitions of the model is the method of choice. But since the size of the state space grows exponentially in the number of model variables, this method is very limited in its applicability. For larger models sampling methods can be used to get some information about model dynamics. There are several existing sophisticated software tools available that allow users to analyze and simulate discrete networks, focused on a particular model type. These tools use a variety of computational and analytical tools for analysis purposes, with a range of different user interfaces; see, e.g., [10-16]. They will be discussed in detail in a later section.

The software tool introduced in this paper, *Analysis of Dynamic Algebraic Models (ADAM) *complements existing software packages in several ways. By translating models into the rich mathematical framework of polynomial dynamical systems over a finite number system, we can bring to bear a variety of theoretical results, computational algorithms, and available software packages from computer algebra and computational algebraic geometry on the analysis of model dynamics. For this purpose we provide implemented algorithms that import models created in with other packages, so that the user does not need to learn a new mathematical framework [17,18]. The basic computational workhorse underlying our software tool is the (symbolic) solution of systems of (nonlinear) polynomial equations over a finite number system. This is a well-studied problem in computer algebra and sophisticated algorithms are implemented for this purpose, which we make use of. An efficient computational implementation results in the ability to analyze model dynamics for quite large discrete models without having to resort to heuristic algorithms. We offer *ADAM *as a web service, avoiding the problems associated with software downloads and different computational platforms.

## Results and Discussion

In this manuscript, we present the web-based tool *ADAM*, Analysis of Dynamic Algebraic Models [19], a tool to study the dynamics of a wide range of discrete models. *ADAM *provides efficient analysis methods based on mathematical algorithms as a web-based tool for several different input formats, and it makes analysis of complex models accessible to a larger community, as it is platform independent as a web-service and does not require understanding of the underlying mathematics. *ADAM *is the successor to DVD, Discrete Visualizer of Dynamics [20], a tool to visualize the temporal evolution of small polynomial dynamical systems.

As the underlying computational approach, we propose a novel method to identify attractors of a discrete model. This method relies on the fact that many discrete models can be translated into the algebraic framework of polynomial dynamical systems. Using these polynomials, one can construct a system of polynomial equations, such that its solutions correspond to fixed points or limit cycles. Thus, the problem of identifying attractors becomes equivalent to solving a system of polynomial equations over a finite field. This is a long-studied problem in computer algebra, and can usually be solved efficiently by using Gröbner basis methods [21]. We emphasize that this method is not a new mathematical algorithm to solve polynomial equations, but a novel approach to the analysis of discrete dynamical systems that uses a novel encoding of the periodic points of such a system as the solutions of polynomial systems derived from the model when expressed in the algebraic framework. *ADAM *allows users unfamiliar with polynomial dynamical systems or Gröbner bases to benefit from this efficient algorithm. We tested the method on several examples and had an average run time of less than one second, comparable to the performance of other software tools; and we were able to identify limit cycles of systems with more than 32 variables in less than one second.

In addition to providing access to mathematical theory for efficient analysis, algebraic models are a unifying framework and systematic approach for several model types. This allows for an effective comparison of heterogeneous models, such as a Boolean network model and an agent-based model. For community integration in the biological sciences, *ADAM *contains a model repository of previously published models available in *ADAM *specific format [22]. This allows new users to familiarize themselves quickly with *ADAM *and to validate and experiment with existing models. In the following section, we discuss general features of *ADAM *briefly and explain new features in more detail.

### General Features of ADAM

*ADAM *is a tool for analyzing different types of discrete models. It automatically converts discrete models into polynomial dynamical systems, that is, time and state discrete dynamical systems described by polynomials over a finite field (see Appendix A.1 for definition and example). The dynamics of the models is then analyzed by using various computational algebra techniques. Even for large systems, *ADAM *computes key dynamic features, such as steady states, in a matter of seconds. *ADAM *is available online and free of charge. It is platform independent and does not require the installation of software or a computer algebra system.

*ADAM *translates the following inputs into (probabilistic) polynomial dynamical systems and can then analyze them.

• Logical models generated with GINsim [10]

• polynomial dynamical systems

• Boolean networks

• probabilistic polynomial dynamical systems, probabilistic Boolean networks (PBN) [6].

*ADAM *also translates Petri nets generated with Snoopy and we plan to implement analysis methods for Petri nets in future versions.

*ADAM*'s main application is the analysis of the dynamic features of a model, which includes the identification of stable attractors. These are either steady states, i.e., time-invariant states, or limit cycles, i.e., time-invariant sets of states. *ADAM *is capable of identifying all steady states and limit cycles of length up to a user-specified length *m*. The process of finding long limit cycles is quite slow for large models. However, in biological models limit cycles are likely to be short, so that *m *can be chosen to be small in general, i.e., less than 10.

The temporal evolution of the model can be visualized by the *state transition graph*, the directed graph of all possible states and edges indicating their transitions, also called the *state space*. For small enough models, i.e., less than eleven variables, *ADAM *generates a graph of the complete state space; for larger models, *ADAM *uses algebraic algorithms to determine dynamic properties. Independent of network size, *ADAM *generates a *wiring diagram*. The wiring diagram, also known as *dependency graph*, shows the static relationship between the variables. All edges in *ADAM*'s wiring diagrams are functional edges, that is, there exists at least one state such that a change in the input variable causes a change in the output variable (see Appendix A.2 for more details). This means that *ADAM *determines all non-functional edges, which is oftentimes of interest.

With *ADAM*, one can also study the temporal evolution of user-specified initial states. The trajectory of a state describes the state's evolution, and it can be computed by repeatedly applying the transition function until an attractor is reached.

All of these features can be computed assuming synchronous updates or sequential updates according to an update-schedule specified by the user. Note that the steady states are the same independent of the update schedule. This is due to the fact that updating any variable at a steady state does not change its value. It is irrelevant for a steady state analysis whether updates are considered to happen sequentially or simultaneously.

For probabilistic networks, i.e., models in which each variable has several choices of local update rules, *ADAM *can generate a graph of all possible updates. This means that states in the phase space can have out-degree greater than one, since different transitions are possible. *ADAM *can find all true steady states, in the context of probabilistic networks, meaning all states that are time-invariant independent of the choice of update function. For further information of probabilistic networks, see [6].

For Boolean networks, *ADAM *calculates all functional circuits (see Appendix A.2). Positive functional circuits are a necessary condition for multi-stationarity. For a certain class of Boolean networks, namely conjunctive/disjunctive networks, *ADAM *computes a complete description of the phase space as described in [23]. For further details on conjunctive networks, see Appendix B.2.

In summary, *ADAM *can generate the following outputs.

• wiring diagram

• phase space for small models

• steady states (for deterministic and probabilistic systems)

• limit cycles of specified length *m*

• trajectories originating from a given initial state until a stable attractor is found

• dynamics for synchronous or sequential updates

• functional circuits for Boolean networks

• a complete description of the phase space for conjunctive/disjunctive networks.

### Applications

We show how to use *ADAM *on a well-understood model of the expression pattern of the segment polarity genes in Drosophila melanogaster. Albert and Othmer developed a model for embryonic pattern formation in the fruit fly Drosophila melanogaster [24]. Their Boolean model consists of 60 variables, resulting in a state space with more than 10^18 ^states. They analyze the model for steady states by manually solving a system of Boolean equations. They also analyze the temporal evolution of a specific initial state corresponding to the wild type expression pattern by repeatedly applying the Boolean update rules until a steady state is found. The update schedule of the model is synchronous with the exception of activation of SMO and the binding of PTC to HH (activation of PH), which are assumed to happen instantaneously. This can be accounted for by substituting the equations for SMO and PH into the update rules for other genes and proteins, rather than using SMO and PH themselves.

To analyze the model, we first rename the variables in the Boolean rules given in [24] such as wg_*i *_or SLP_*i *_to *x*_1 _... *x*_60_, to standardize their format. The variables *x_i _*and their corresponding genes are listed in Table 1. Then we use *ADAM*: the model type is *Polynomial Dynamical Systems*, the number of states in a Boolean model is 2, representing 'present' or 'absent'. One can choose *Boolean*, and enter the Boolean rules in the text-area or upload a text file with the Boolean rules. Alternatively, one can first convert the Boolean rules to polynomials over , and enter the polynomials with the choice *Polynomial*. The file with the polynomial equations for the model can be accessed at [22].

**Table 1:** Correspondence of genes and variable names

Cell 1	SLP x1	wg x2	WG x3	en x4	EN x5	hh x6	HH x7	ptc x8	PTC x9	PH x10	SMO x11	ci x12	CI x13	CIA x14	CIR x15
Cell 2	SLP x16	wg x17	WG x18	en x19	EN x20	hh x21	HH x22	ptc x23	PTC x24	PH x25	SMO x26	ci x27	CI x28	CIA x29	CIR x30

Cell 3	SLP x31	wg x32	WG x33	en x34	EN x35	hh x36	HH x37	ptc x38	PTC x39	PH x40	SMO x41	ci x42	CI x43	CIA x44	CIR x45

Cell 4	SLP x46	wg x47	WG x48	en x49	EN x50	hh x51	HH x52	ptc x53	PTC x54	PH x55	SMO x56	ci x57	CI x58	CIA x59	CIR x60

Genes and proteins in [24] and their corresponding variable names *x*_1_,..., *x*_60_.

**Figure 1:**
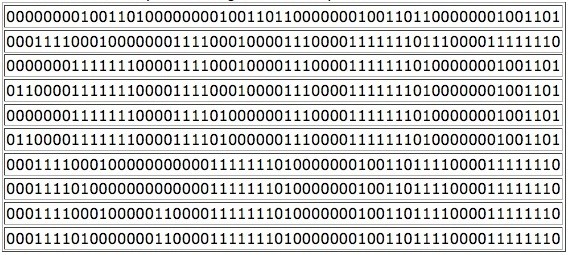
***ADAM*: Analysis of steady states of Drosophila model**. Each row in the table corresponds to a stable attractor. Attractors are written as binary strings, where 0 represents non-expression of a gene (or low concentration of a protein), and 1 expression (or high concentration). Steady states of Drosophila Melanogaster as found with *ADAM*.

The rules in the model file are specified in *Polynomial *form. Once the polynomials are uploaded, we need to set the *Analysis *type. The model with 60 variables is too complex for exhaustive enumeration, and we choose *Algorithm*. This means that instead of exhaustive enumeration of the state space, analysis of the dynamics is done via computer algebra by solving systems of equations. In *Options*, we set *Limit cycle length *to 1 because we are interested in the steady states, i.e., time-invariant states. We chose *Synchronous *as updating scheme. Once these choices have been made, we obtain the steady states by clicking *Analyze. ADAM *returns a link to the *wiring diagram *(or *dependency graph*), which captures the static relations between the different variables. In addition, *ADAM *returns the number of steady states and the steady states themselves: see Figure 1. These steady states are identical to those found in [24], half of which have been observed experimentally.

Each row in the table in Figure 1 corresponds to a stable attractor. Attractors are written as binary strings, where 0 represents non-expression of a gene (or low concentration of a protein), and 1 expression (or high concentration); e.g., the binary string(1)

corresponds to the genes (and proteins) being expressed (or present in high concentration) in four cells from anterior to posterior compartments (compartment 1 to 4). The string can be translated back to a list of genes that are expressed in this stable attractor; see Table 2. This is the steady state obtained in [24] when starting the system with an initial state representing the experimental observations of stage 8 embryos. *ADAM *can also generate trajectories for a given initial state. For example, we can choose the initial state that was used in [24] representing stage 8 embryos. Again, we enter *Polynomial Dynamical Systems *with 2 as the number of states and upload the polynomials describing the model. Instead of *Algorithms*, we now choose *Simulation*. Since we are not interested in the number of steady states or the complete phase space, but in a single trajectory originating from a specific initial state, we choose *One trajectory starting at an initial state *as the simulation option. We enter the state corresponding to the initial state shown in table 3 as a binary string:(2)

**Table 2:** Genes and proteins present in steady state

compartment 1	en, EN, hh, HH, SMO
compartment 2	ptc, PTC, PH, SMO, ci, CI, CIA

compartment 3	SLP, PTC, ci, CI, CIR

compartment 4	SLP, wg, WG, ptc, PTC, PH, SMO, ci, CI, CIA

Genes and proteins present in steady state corresponding to binary string (1).

**Table 3:** Genes and proteins present in initial state

compartment 1	en, hh
compartment 2	ptc, ci

compartment 3	SLP, ptc, ci

compartment 4	SLP, wg, ptc, ci

Genes and proteins present in initial state corresponding to binary string (2).

**Figure 2:**
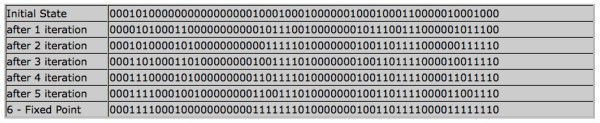
***ADAM*: Trajectory of Drosophila model**. Temporal evolution of given initial state until steady state is reached.

By clicking *Analyze*, we obtain the temporal evolution of this particular state until it reaches a steady state; see Figure 2. As predicted in [24], the steady state is the state corresponding to the state shown in Table 2. To summarize, *ADAM *correctly identified the steady states in less than one second. All steady states have been determined previously in [24] by labor-intensive manual investigation of the system.

Furthermore, we used *ADAM *to verify that there are no limit cycles of length two or three. The model has not been analyzed previously for limit cycles. The absence of two- and three-cycles strengthens confidence in the model, since oscillatory behavior has not been observed experimentally. Computations for limit cycles of length greater than three have not been conducted, as composing the system several times with itself is computationally complex. The model file in *ADAM *format can be accessed at [22].

### Benchmark Calculations

**Figure 3:**
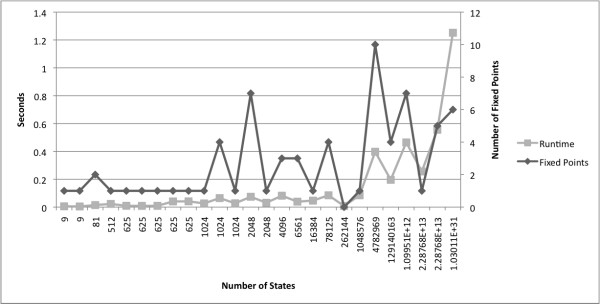
**Runtime of steady state calculations of several logical models from **[25]. Executed on a 2.7 GHz computer.

We analyzed logical models available in the GINsim model repository [25] as of August 2010. The repository consists of models in GINsim XML format previously published in peer-reviewed journals. We converted all but two models into polynomial dynamics systems. For these 26 models we computed the steady states. All calculations finished in less than 1.5 seconds; see Figure 3.

In addition to the published models in [25], we analyzed randomly generated networks that have the same structure that we expect from biological systems, namely sparse, i.e., while the number of nodes in a biological network may be quite large, each node is affected only by a small number of other nodes, and robust, i.e., small number of attractors. We tested a total of 50 networks with 50-150 nodes (10^15 ^- 10^45 ^states) and an average of average in-degrees of 1.6848. The steady state calculations took less than half a second for each network on a 2.7 GHz computer.

### Comparison to Other Systems

In this section, we describe the functionality of several state-of-the-art software tools for the analysis of discrete models of biological systems. They are all capable of identifying steady states and limit cycles by exhaustive enumeration of the state space for small models (less than 32 variables) [10,11,13-16]. For larger models, GINsim is capable of analyzing models for steady states, and several tools provide heuristic analysis methods. None of them identifies limit cycles deterministically for models with more than 32 variables. It is important to stress that ADAM provides a web-interface and does not require local installation as all the other tools do, which makes them less accessible to users. Table 4 summarizes the features of the different software tools, which we will now explain in detail.

**Table 4:** Software Comparison

	Steady State Analysis	Limit Cycle Analysis	Input Format	System Requirements
ADAM	Yes^‡^	Yes^◊^	Boolean (or polynomial) functions Logical Models (GINsim)	None, web based

GINsim	Yes^‡^	For small models	Parameters (non-zero truth tables) Logical Model	Java virtual machine^○^

BoolNet R package	For small ^† ^models	For small ^† ^models	Boolean functions	R statistics software

DDLab	For small models	For small models	Logical tables	*○*

BN/PBN Matlab Toolbox	For small models	For small models	Logical tables	Matlab

Comparison of different software tools regarding attractor analysis: ‡ less than 1 second on published gene regulatory networks with up to 72 variables; ◊ only for short limit cycles; † heuristic methods are available for larger networks; ○ installation necessary, available for common operating systems.

*GINsim *(Gene Interaction Network simulation) is a package designed for the analysis of gene regulatory networks [10]. As input, it accepts logical models. Logical models are an extension of Boolean models; they consist of similar switch-like rules, but allow for a finer data discretization with more than two states per variable, e.g., *low*, *medium*, and *high*. Logical models can be updated synchronously or asynchronously. For the latter, the temporal evolution of a logical model is non-deterministic because the variables are updated randomly in an asynchronous fashion. In either case, updates of every variable are *continuous*, meaning that no variable changes its value by more than one unit in one time-step, see section *Remarks about Logical Models *for a detailed discussion.

GINsim provides algorithms that use binary decision diagrams (BDD) for the determination of steady states [7]. Analysis of limit cycles is executed by simulating every trajectory, i.e., generating the complete state space, called state transition graph in GINsim, and therefore limited by network size. We tested GINsim on logical models with up to 72 variables; determining the steady states took less than one second. More complex logical networks were not available to us.

*BoolNet R package *provides methods for inference and analysis of synchronous, asynchronous, and probabilistic Boolean networks [11]. It is a package for the free statistics software *R*, and it is run via the *R *command-line. It is helpful, if the user is already familiar with *R*. Steady state analysis is implemented as exhaustive search of the state space, heuristic search, random walk, or Markov Chain analysis [6].

Non-heuristic analysis is limited to networks with 29 variables. For larger networks, steady states can be inferred heuristically, which does not guarantee that all steady states are identified.

*DDLab *is an interactive graphics software for discrete models, including cellular automata, Boolean and multi-valued networks [13]. As it is mainly a visualization tool, analysis is based on exhaustive enumeration of the state space, and model size is limited to 31 variables.

*BN/PBN Toolbox *is a toolbox written in *Matlab *[14]. It uses the state transition matrix to compute attractors. Statistics for networks with more than 27 variables cannot be computed ("Maximum variable size allowed by the program is exceeded"). In addition to analyzing deterministic Boolean networks, the toolbox can analyze probabilistic Boolean networks and calculate statistics such as numbers and sizes of attractors, basins, transient lengths, Derrida curves, percolation on 2-D lattices, and influence matrices.

#### Remarks about Logical Models

In this manuscript, we distinguish between three different update types: synchronous, sequential according to an update schedule, and asynchronous. *ADAM *allows for synchronous or sequential updates according to a given update schedule. In models with synchronous updates, all variables are updated simultaneously at every time step. In models with sequential updates according to an update schedule, all variables are updated at every time-step in the order given by the schedule. Both these model types are deterministic.

In models with asynchronous updates, as is common for logical models, one variable is updated at random at every time step, which results in a non-deterministic model. Models with sequential updates according to an update schedule produce dynamics that are different from that of models with asynchronous updates, i.e., logical models.

In GINsim, all models are *continuous *in the sense that at each time-step, each variable increases or decreases by at most one unit. Though logical models are discrete, there are no jumps skipping intermediate states. For example, in a model with three states, low, medium, and high, no variable can drop from high to low in a single update step. This interpretation is different from the common meaning of continuous, which usually refers to models of ordinary or partial differential equations. The parameters entered in GINsim specify the target value towards which the variable changes, i.e., the value increases by one, decreases by one, or remains constant if the target value is larger, smaller, or equal than the initial value, respectively. The state transition graph generated with *ADAM *might differ from the state transition graph generated in GINsim. To obtain the exact same state transition graph, every variable in the logical model must contain an explicit self-loop, and all parameters must be entered such that the target value differs by at most one from the value of the variable to be updated. Any logical model can be specified in this way without changing its state transition graph. Boolean models are always continuous.

In multi-valued logical models, variables can have different maximum values. In an algebraic model, all variables are defined over the same algebraic field, i.e., have the same maximum value. When a multi-valued logical model is translated into an algebraic model, extraneous states might be introduced such that all variables are defined over the same field. An example of such an extension is given in Table 5, the extra states are the states in the last row, which are given the same values as the states above to extend the model in a meaningful way. The extra states should be ignored when analyzing the dynamics. For more details, see [17].

**Table 5:** Multi-valued models

**next state of *x***_**2**_	**low *x***_**2**_	**medium *x***_**2**_	**high *x***_**2**_
*x*_1 _absent	low *x*_2_	medium *x*_2_	high *x*_2_

*x*_1 _present	medium *x*_2_	high *x*_2_	high *x*_2_

extension *x*_1 _present	medium *x*_2_	high *x*_2_	high *x*_2_

Updates for variable *x*_2 _in a logical model, where *x*_2 _depends on *x*_1 _and itself. The states 0 and 1 represent absent and present for the Boolean variable *x*_1_; 0, 1, and 2 represent low, medium, and high for the multi-valued variable *x*_2_. The last row is introduced in the polynomial dynamical system such that all variables are defined over . The extra states (2, 0), (2, 1), (2, 2) in the state space should be ignored when interpreting the dynamics.

### Architecture

*ADAM *is available as a web-based tool that does not require any software installation. *ADAM*'s user interface is implemented in HTML. We use JavaScript to generate a dynamic website that adapts as the user makes various choices. This simplifies the process of entering a model. For example, after defining the model type, i.e., Polynomial Dynamical System, Probabilistic Network, Petri net, and Logical Model the next line changes to the number of states, *k*-bound, or nothing, appropriately. Input can be entered directly into the text area, or uploaded as a text document.

All mathematical algorithms are programmed in Macaulay2 [26]. Macaulay2 is a powerful computer algebra system. The routines for which fast execution is crucial are implemented in C/C++ as part of the Macaulay2 core. Logical Models and Petri nets in XML format are parsed using Ruby's XmlSimple library. The interplay between HTML and Macaulay2 is also programmed in Ruby.

Output graphs are generated with Graphviz's *dot *command. When *Simulation *is chosen as analysis method, Graphviz's *ccomps - connected components filter for graphs *is used to count the connected components. A Perl script directs the execution of the Graphviz commands.

### Model Repository

A model repository is part of the *ADAM *website [22]. The repository consists of a collection of several previously published models in *ADAM *format. The models are extracted from publications, and rewritten in *ADAM *specific format, i.e., all variables are renamed to *x_i _*and the update rules from the original publication are reformulated as Boolean rules or polynomials. The central repository with models in a unified format allows for quick verification and experimentation with published models. By changing parameters or initial states, users can gain a better understanding of the models.

New users can also use the repository to quickly familiarize themselves with the main functionalities of *ADAM*. In addition to the model itself, the database entries contain a short summary of the biological system and relevant graphs, together with an analysis of dynamic features determined by *ADAM *and their biological explanation. The repository is work in progress by researchers from several institutions generating more entries for the repository. We invite all interested researchers to submit their models. Because of their intuitive nature, discrete models are an excellent introduction to mathematical modeling for students of the life sciences. *ADAM*'s model repository is a great starting point to familiarize students with the abstraction of discrete models such as Boolean networks.

## Conclusions

Discrete modeling techniques are a useful tool for analyzing complex biological systems and there is a need in the biological community for easy to use analysis software. *ADAM *provides efficient methods as a web-based tool and will allow a larger community to use complex modeling techniques, as it is platform independent and does not require the user to understand the underlying mathematics. Upon translating discrete models, such as logical networks, Petri nets, or agent-based models into algebraic models, rich mathematical theory becomes available for model analysis, e.g., for steady state and limit cycle analysis.

After extensive experimentation with both discrete models arising in systems biology and randomly generated networks, we found that the algebraic algorithms presented in this manuscript are fast for sparse systems with few attractors, a structure maintained by most biological systems. All algorithms have been included in the software package *ADAM *[19], which is user-friendly and available as a free web-based tool. *ADAM *is highly suitable to be used in a classroom as a first introduction to discrete models because students can use it without going through an installation process.

*ADAM *provides methods to analyze the key dynamic features, such as steady states and limit cycles, for large-scale (probabilistic) Boolean networks and logical models. *ADAM *unifies different modeling types by providing analysis methods for all of them and thus can be used by a larger community.

We hope to expand *ADAM *to a more comprehensive Discrete Toolkit which incorporates new analysis methods, better visualization, and automatic conversion for more model types. We also hope to analyze controlled algebraic models and expand theory to stochastic systems.

## Methods

Logical models, Petri nets, and Boolean networks are converted automatically into the corresponding polynomial dynamical system as described in [17], so that algorithms from computational algebra can be used to analyze the dynamics. In polynomial dynamical systems over a finite field, states of a variable are assigned to values in the field, and the update (or transition) rule for each variable is given as a polynomial rather than a Boolean or logical expression. For more details, see Appendix A.1. Using these polynomials, one can construct systems of polynomial equations, such that their solutions correspond to fixed points or limit cycles. Thus, the problem of identifying attractors becomes equivalent to solving a system of polynomial equations over a finite field. This is a long-studied problem in computer algebra, and can usually be solved efficiently by Gröbner basis methods.

Gröbner basis calculation is for polynomial systems what Gauss-Jordan elimination is for linear systems: a structured way to transform the original system to triangular shape without changing its solution space. The triangular shape of the resulting systems allows for stepwise retrieval of the solutions of the system. For a more in depth discussion of Gröbner bases, see for example [21].

In the worst case, computing Gröbner bases for a set of polynomials has complexity doubly exponential in the number of solutions to the system. However, in practice, Gröbner bases are computable in a reasonable time. It has been suggested, that in robust gene regulatory networks genes are regulated by only a handful of regulators [27]. Thus, the polynomial dynamical systems representing such biological networks are sparse, i.e., each function depends only on a small subset of the model variables. From our experience, a Gröbner basis calculation for sparse systems with few attractors, a structure common for biological systems, is actually quite fast.

### A Mathematical Background

#### A.1 Polynomial Dynamical Systems

To be self-contained, we briefly explain polynomial dynamical systems and their key features. A polynomial dynamical system(PDS) [28] over a finite field *k *is a function

with coordinate functions *f_i _*∈ *k*[*x*_1_,..., *x_n_*], the ring of polynomials in the variables *x_i_*, with coefficients in *k*. Iteration of *f *results in a time-discrete dynamical system. A PDS can be used to describe the dynamic behavior of a biological system: every variable *x_i _*corresponds to a biological substrate, for example a protein or gene, and the polynomials *f_i _*describe the evolution of *x_i _*depending on the previous state of the variables *x*_1_,..., *x_n_*.

PDS have several dynamic features of biological relevance. These include the number of components, component sizes, steady states, limit cycles, and limit cycle lengths.

**Example **Let  and *f *= (*f*_1_, *f*_2_, *f*_3_):  with

**Figure 4:**
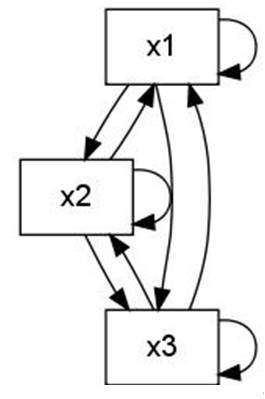
**Wiring diagram: static relationship between variables**.

**Figure 5:**
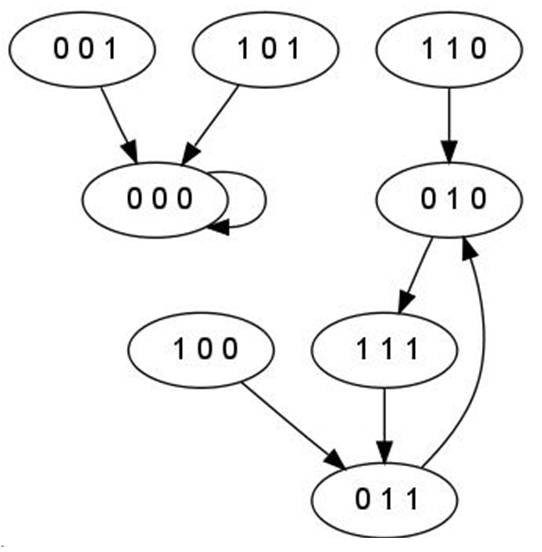
**Phase space: temporal evolution of the system**.

The wiring diagram of *f*, which shows the static interaction of the three variables, is depicted in Figure 4 along with its phase space in Figure 5. The state transition graph shows the temporal evolution of the system. Each state is represented as a vector of the values of the three variables (*x*_1_, *x*_2_, *x*_3_). The PDS described by *f *has two stable attractors: a steady state, (000), and a limit cycle of length three, consisting of the states (010), (111), and (011).

A probabilistic PDSover a finite field *k *is a collection of functions

together with a probability distribution for every coordinate that assigns the probability that a specific function is chosen to update that coordinate. The coordinate functions *f_i, j _*are elements in *k*[*x*_1_,..., *x_n_*]. Probabilistic PDS, specifically Boolean probabilistic networks (PBN), have been studied extensively in [6]. *ADAM *analyzes probabilistic PDS. It can simulate the complete state transition graph for sufficently small models, by generating every possible transition and labeling the edge with its probability according to the distribution. If no distribution is given, *ADAM *assumes a uniform distribution on all functions. For large networks, *ADAM*'s *Algorithm *choice computes steady states of probabilistic networks.

#### A.2 Functional Edges

An edge in the wiring diagram from *x_i _*to *x_j _*is considered functional, if there exists a state  such that , where *a *and *b *are values for *x_i_*, in other words, if there is at least one state, such that changing only *x_i _*but keeping all other values fixed, changes the next state of *x_j_*. In *ADAM*, all edges in the wiring diagram are functional. For Boolean networks, *ADAM *identifies all functional elementary circuits. An elementary circuit is a finite closed path in the wiring diagram in which all the nodes are distinct. The existence of functional circuits is a necessary condition for multi-stationarity and limit cycles. For a further discussion of functional circuits, see [7]. For multivalued networks, circuit analysis has not yet been implemented.

### B Algorithms

#### B.1 Analysis of stable attractors

Every attractor in a PDS is either a steady state or a limit cycle. For small models, *ADAM *determines the complete phase space by enumeration, for large models, *ADAM *computes steady states and limit cycles of a given length. A state is a steady state, if it transitions to itself after one update of the system. A state is part of a limit cycle of length *m*, if, after *m *updates, it results in itself. Any steady state of a PDS satisfies the equation *f*(*x*) = *x*, as no coordinate of *x *is changing as it is updated. Similarly, states of a limit cycle of length *m *satisfy the equation *f ^m^*(*x*) = *x. ADAM *computes all steady states by solving the system *f_i_*(*x*) *- x_i _*= 0 for *i *∈ {1,..., *n*} simultaneously. To efficiently solve the resulting systems of polynomial equations, we first compute the Gröbner basis in lexicographic order for the ideal generated by the equations. Choosing a lexicographic order allows to easily obtain the solutions [21]. We use the Gröbner basis algorithms distributed with Macaulay2, version 1.3.1.1, and found that for quotient rings over a finite field the implementation 'Sugarless' is more efficient than the default algorithm with 'Sugar' [26,29]. For limit cycles of length *m*, the solutions of *f^m^*(*x*) = *x *are found and then grouped into cycles, by applying *f *to each of the solutions.

**Example **Fixed points of the system shown in the example in A.1 are solutions in  of the system *f*(*x*) = *x*:

The only solution to this systems is the point (*x*_1_, *x*_2_, *x*_3_) = (0, 0, 0). This is in accordance with the state transition graph depicted in Figure 5: (0, 0, 0) is the only steady state. To investigate limit cycles of length two, one has to look at the system *f *^2^(*x*) = *x*,

Again, (0, 0, 0) is the only solution, which means that there are no limit cycles of length two. Investigating *f *^3^(*x*) = *x*,

results in the solutions (0, 0, 0), (0, 1, 0), (0, 1, 1), (1, 1, 1). (0, 0, 0) is a steady state, and (0, 1, 0), (0, 1, 1), (1, 1, 1) are elements of a limit cycle of length 3. For all *m >*3, *f ^m^*(*x*) = *x *has no solutions, that means the system *f *has exactly two attractors, a steady state a a limit cycle of length 3.

#### B.2 Conjunctive/Disjunctive Networks

Some classes of networks have a certain structure that can be exploited to achieve faster calculations. Jarrah et al. show that for conjunctive (disjunctive) networks key dynamic features can be found with almost no computational effort [23]. Conjunctive (respectively disjunctive) networks consist of functions using only the AND (respectively OR) operator. *ADAM *comes with an implementation of this algorithm to analyze dynamics in the case of conjunctive (disjunctive) networks. Currently, this option is only implemented for networks with strongly connected dependency graphs.

## Authors' contributions

FH led the algorithm and software development. BG, MB, and RM implemented the user interface and attractor analysis, executed benchmarking calculations, and drafted the initial manuscript under FH's direction. AV implemented the translation for logical algorithms to PDS used by *ADAM*. GB participated in the software design effort and algorithm development. RL conceived of the study, provided overall leadership of the project, and secured funding for it. He also contributed to the writing and editing of the manuscript. All authors read and approved the final manuscript.
